# Brompton Hospital for Consumption

**Published:** 1906-12-01

**Authors:** 


### BROMPTON HOSPITAL FOR CONSUMPTION.

### FINANCIAL CONSEQUENCES OF SANATORIA.

A Quarterly Court of Governors of this excellent charity was held in the Board Room of the hospital on November 23, Major-General the Right Hon. Lord Cheylesmore, C.V.O., Chairman of the Committee of Management, presiding.

It was announced that Dr. Horton-Smith Hartley, M.V.O., had been appointed a physician to the hospital; Dr. Cecil Wall an assistant physician; Dr. Dundas Grant as surgeon in charge of the throat department; and Dr. Arthur C. Inman as superintendent of the Clinical Laboratory.

The Chairman referred to the loss, through death, of Colonel the Hon. N. Dalrymple Hamilton, a valued member of the Board of Management, and of Dr. Gustave Schorstein, one of the physicians to the hospital.

He also touched upon the loss of revenue occasioned this year through the serious falling-off in legacies, and that consequently an urgent appeal for £5,000 to meet current liabilities was now being made. The addition of the Sanatorium and Convalescent Home, containing 108 beds, to the work of the hospital had greatly augmented the difficulty in raising the necessary funds to carry on its work, for since in the past the income of the hospital had been sufficient for its needs the addition of the sanatorium had necessitated an increased expenditure of at least £8,000 per annum, the whole of which could only be met by voluntary benefactions.

